# Cleavage of Host Cytokeratin-6 by Lysine-Specific Gingipain Induces Gingival Inflammation in Periodontitis Patients

**DOI:** 10.1371/journal.pone.0117775

**Published:** 2015-02-17

**Authors:** Salunya Tancharoen, Takashi Matsuyama, Ko-ichi Kawahara, Kenji Tanaka, Lyang-Ja Lee, Miho Machigashira, Kazuyuki Noguchi, Takashi Ito, Takahisa Imamura, Jan Potempa, Kiyoshi Kikuchi, Ikuro Maruyama

**Affiliations:** 1 Department of Pharmacology, Faculty of Dentistry, Mahidol University, Bangkok, Thailand; 2 Department of Periodontology, Kagoshima University Graduate School of Medical and Dental Sciences, Kagoshima, Japan; 3 Laboratory of Functional Foods, Department of Biomedical Engineering, Osaka Institute of Technology, Osaka, Japan; 4 Membrane Protein and Ligand Analysis Center, Protosera Inc., Amagasaki, Japan; 5 Department of Systems Biology in Thromboregulation, Kagoshima University Graduate School of Medical and Dental Science, Kagoshima, Japan; 6 Department of Molecular Pathology, Faculty of Life Sciences, Kumamoto University, Kumamoto, Japan; 7 Department of Periodontics, Endodontics and Dental Hygiene, University of Louisville School of Dentistry, Louisville, Kentucky, United States of America; 8 Department of Microbiology, Faculty of Biochemistry, Biophysics and Biotechnology, Jagiellonian University, Kraków, Poland; 9 Department of Physiology, Kurume University School of Medicine, Fukuoka, Japan; Boston University, UNITED STATES

## Abstract

**Background/Purpose:**

Lysine-specific gingipain (Kgp) is a virulence factor secreted from Porphyromonas gingivalis (P. gingivalis), a major etiological bacterium of periodontal disease. Keratin intermediate filaments maintain the structural integrity of gingival epithelial cells, but are targeted by Kgp to produce a novel cytokeratin 6 fragment (K6F). We investigated the release of K6F and its induction of cytokine secretion.

**Methods:**

K6F present in the gingival crevicular fluid of periodontal disease patients and in gingipain-treated rat gingival epithelial cell culture supernatants was measured by matrix-assisted laser desorption/ionization time-of-flight mass spectrometer-based rapid quantitative peptide analysis using BLOTCHIP. K6F in gingival tissues was immunostained, and cytokeratin 6 protein was analyzed by immunofluorescence staining and flow cytometry. Activation of MAPK in gingival epithelial cells was evaluated by immunoblotting. ELISA was used to measure K6F and the cytokines release induced by K6F. Human gingival fibroblast migration was assessed using a Matrigel invasion chamber assay.

**Results:**

We identified K6F, corresponding to the C-terminus region of human cytokeratin 6 (amino acids 359–378), in the gingival crevicular fluid of periodontal disease patients and in the supernatant from gingival epithelial cells cultured with Kgp. K6F antigen was distributed from the basal to the spinous epithelial layers in gingivae from periodontal disease patients. Cytokeratin 6 on gingival epithelial cells was degraded by Kgp, but not by Arg-gingipain, P. gingivalis lipopolysaccharide or Actinobacillus actinomycetemcomitans lipopolysaccharide. K6F, but not a scrambled K6F peptide, induced human gingival fibroblast migration and secretion of interleukin (IL)-6, IL-8 and monocyte chemoattractant protein-1. These effects of K6F were mediated by activation of p38 MAPK and Jun N-terminal kinase, but not p42/44 MAPK or p-Akt.

**Conclusion:**

Kgp degrades gingival epithelial cell cytokeratin 6 to K6F that, on release, induces invasion and cytokine secretion by human gingival fibroblasts. Thus, Kgp may contribute to the development of periodontal disease.

## Introduction

Periodontal disease (PD) is caused by irritation of the periodontal tissues by a multitude of bacterial species. When coupled with the host defense mechanism, this damages the periodontium and, if left untreated, can result in tooth loss [1]. PD is a persistent inflammatory disease, characterized by massive inflammatory cell infiltration into the gingival tissues, increased crevicular fluid production and apical migration of junctional epithelial cells into the surrounding connective tissue, leading to a loss of connective tissue and alveolar bone [2,3]. *Porphyromonas gingivalis (P*. *gingivalis)* is a major periodontal pathogenic bacterium whose virulence is mediated in part by proteases of the gingipain family [4,5]. Gingipains are produced by two genes that encode Arg-specific proteases (RgpA and RgpB) and another that encodes a Lys-specific protease (Kgp). Of the three gingipains in human plasma, Kgp is the most potent fibrinogen/fibrin-degrading enzyme and is involved in bleeding in diseased gingiva [4]. In contrast to Arg-gingipain, Kgp is not inhibited by hemin, suggesting that its role in PD progression is near the cell surface [6]. Kgp has numerous modes of action. It is required initially for *P*. *gingivalis* adhesion to the host tissue *via* its adhesion domains, and possibly *via* the related domains of hemagglutinin A (HagA) that bind to epithelial cells [7]. Kgp also cleaves hemoglobin [6], haptoglobin and hemopexin, ultimately releasing heme, which promotes bacterial growth [8]. Third, RgpA-Kgp proteinase complexes trigger an inflammatory response by deregulating the cytokine network. At low concentrations, these complexes induce proinflammatory cytokine secretion in gingival tissue, whereas at high concentrations they attenuate proinflammatory mediators by inducing cellular apoptosis [9]. Finally, Kgp induces periodontal bone loss by degrading osteoprotegerin [10]. This evidence suggests that heme acquisition and regulation of inflammatory processes underpin the action of Kgp in promoting periodontal tissue destruction.

Keratins are the major structural proteins of vertebrate epithelial cells and form an intricate cytoplasmic network of 10-nm intermediate filaments. This network is required for maintenance of epithelial cell integrity [11] and protection of epithelial cells from mechanical and non-mechanical stress and injury [12]. Keratins are encoded by a large family of genes clustered at two divergent chromosomal sites: 17q21.2 (type I keratins, except keratin 18) and 12q13.13 (type II keratins, including keratin 18) [13]. Epithelial cell keratins consist of non-covalently-associated type I (keratin 9–keratin 20) and type II (keratin 1–keratin 8) keratins. Gingival keratinocytes express different keratin pairs at their various differentiation states. The basal proliferative layers of all oral epithelia express keratin 5/keratin 14 and keratin 19. The suprabasal differentiating layers of keratinized (cornified) epithelia express keratin 1 and keratin 10, while the suprabasal epithelial cells of the hard palate and gingiva express keratin 6, keratin 16, and keratin 7 [14]. Keratin-6a and -6b are basic type II intermediate filament proteins constitutively expressed in epithelial appendages [15], and are inducibly expressed in other types of epithelia when subjected to disease or environmental challenge (e.g., after wounding or treatment with phorbol esters) [16] Recent studies demonstrated that keratin filaments participate in the inflammatory network. For instance, loss of keratin 8 causes hyperplasia and colitis with increased T-cell recruitment and upregulation of T-helper (Th) 2 cytokines [17]. Additionally, keratin 6 mutations cause inherited genodermatosis, cell migration and delayed wound healing [18]. Dominant-negative mutants of keratin-6a experience destruction of the outer root sheath of the hair follicles [19] and skin blistering in the epidermal layer [20]. Furthermore, keratin 6 transcription participates in skin inflammatory reactions induced by interleukin (IL)-1 [21]. Proteomic analysis demonstrated increased expression of keratin 17 in endothelial cells, which may contribute to angiogenesis [22]. Thus, the loss of keratin filaments appears to contribute to the pathophysiology of various human diseases.

In the last decade, differential proteomic analysis has been used to identify target proteins in various diseases. However, the entire set of protease substrates (protein degradome) of Kgp and the role of keratin cleavage by Kgp in the pathogenesis of PD have not been elucidated. We therefore hypothesized that a small keratin 6 fragment (K6F) released by Kgp leaks into the gingival crevicular fluid (GCF) of PD patients and can influence gingival inflammation. To prove this hypothesis, we studied whether this fragment promoted gingival fibroblast migration and/or induced pro-inflammatory cytokine release from gingival fibroblasts *via* mitogen-activated protein kinase (MAPK) signaling. Our study indicates that cleavage of host keratin 6 by Kgp regulates inflammatory- and immune-based processes in oral epithelia and gingival fibroblasts and is involved in PD.

## Materials and Methods

### Ethics Statement

All human samples from patients and healthy volunteers were obtained with the written informed consent of the study participants. Animal care and housing, and any procedures involving animals, were reviewed and approved by the Institutional Animal Care and Use Committee, Kagoshima University, Japan. All experimental protocols were approved by the board members of the Ethics Committee of Clinical Research at Kagoshima University, Japan.

### Study population and periodontal clinical measurements

The study population was divided into two groups: i) chronic PD patients who needed tooth extraction performed for therapeutic purposes (10 females and 10 males; aged 30–65 years) and ii) age-matched control patients who were receiving treatment of periodontal surgery e.g. dental implant surgery or free gingival graft (eight females and three males, with no evidence of PD). All PD patients met the following criteria: (1) moderate/advanced adult periodontitis as defined by a) multiple interproximal probing depths ≥5 mm in each quadrant, b) bleeding on gentle probing, and c) radiographic bone loss; (2) good general health with no history of disease or any medication, including antibiotics during the previous 6 months; and (3) no history of smoking. Healthy participants had pocket depths ≤2 mm, no bleeding on probing and no signs of alveolar bone loss.

### GCF sampling and evaluation

GCF samples were collected from 10 diseased sites per patient in 12 PD patients referred for treatment at the Department of Periodontology, Kagoshima University, Kagoshima, Japan. The samples were taken from untreated diseased sites or from diseased sites with deep pathological pockets persisting after periodontal treatment. GCF samples were also collected from 10 sites per volunteer in 11 healthy volunteers. GCF was acquired by inserting a paper strip (Periopaper; Harco, Tustin, CA, USA) into the gingival crevice for 30 s. Each strip was measured for fluid volume using a Periotron 6000 (IDE Interstate, NY, USA). The strip was then immediately transferred to PBS containing 0.1% bovine serum albumin (BSA) and a cocktail of protease inhibitors (Sigma-Aldrich, St Louis, MO, USA) in a plastic micro-centrifuge tube, frozen within 10 min and stored at −70°C until use. Before the assay, GCF was eluted from the paper strips by soaking each strip in PBS/0.1% BSA/0.05% thimerosal for 18 h at 4°C. Eluted proteins from 10 sites of the same clinical category were pooled for MALDI-TOF mass spectrometry analysis and for ELISA experiment.

### Primary cell cultures

Gingival epithelial cells (GECs) were obtained from 2-week-old Rowett rats. Palatal gingival explants were prepared according to the method described in our previous study [23], with some modification. Briefly, the palatal gingival tissues were resected from rats, placed in tissue culture plates and soaked in Dulbecco's-modified Eagle Medium (DMEM; Sigma-Aldrich, St. Louis, MO, USA) containing 10% fetal bovine serum (FBS). After 2 weeks, GECs were harvested from the culture medium and further cultured in Keratinocyte-Serum Free Medium (Life Technologies, Rockville, MD, USA) supplemented with epidermal growth factor (5 ng/ml) and bovine pituitary extract (30–50 μg/ml). The cells were used for experiments after 4–6 passages. To eliminate the possible side effect of growth factors, all cells were cultured in serum-free media for at least 15 h before treatment. Human gingival fibroblasts (HGFs) were obtained from non-diseased gingiva during periodontal surgery, as described previously [24]. Briefly, the tissues were cultured at 37°C in Dulbecco’s modified Eagle’s medium (DMEM; Sigma-Aldrich, St Louis, MO, USA) supplemented with 10% FBS, 100 U/ml of penicillin G, and 100 μg/ml of streptomycin in a humidified atmosphere at 5% CO_2_. The outgrowing cells were subcultured and used for experiments at passages 4–10.

### Purification and activation of Kgp, HRgpA and RgpB gingipains

Kgp, RgpA and RgpB from *P*. *gingivalis* strain W50 were purified as previously described [25]. Purified gingipains were activated with 10 mM L-cysteine in 0.2 M HEPES buffer (pH 8.0) containing 5 mM CaCl_2_ at 37°C for 8 min and then kept at room temperature. The proteinase was diluted with 50 mM Tris-HCl (pH 7.4) containing 0.1 M NaCl and 5 mM CaCl_2_ immediately before use. Kgp activity was assessed using 0.1 M N-a-acetyl-L-lysine-p-nitroanilide (Bachem AG, Bubendorf, Switzerland) in NaCl/Tris buffer (pH 7.5). The concentration of active Kgp was calculated from the amount of the inhibitor (Tosyl-L-lysine chloromethyl ketone; TLCK) needed for complete inactivation of the protease. To inhibit gingipain activity, Kgp was pretreated for at least 30 min with 10 mM Kgp inhibitor (TLCK) before addition to the cells [26].

### Peptidomic analyses of GCF and rat GEC supernatant by MALDI-TOF mass spectrometry

Matrix-assisted laser desorption/ionization time-of-flight mass spectrometer (MALDI-TOF-MS)-based rapid quantitative peptidomic analysis was performed using the BLOTCHIP-MS method (Protosera, Amagasaki, Japan) as described previously [27,28]. Samples (25 μl) of GCF or rat GEC supernatant were mixed with 30 μl of NuPAGE LDS sample buffer (Life Technologies, Carlsbad, CA, USA). After a brief centrifugation, the supernatants were transferred to new tubes, heated for 10 min at 70°C, and then chilled on ice. A sample (25 μl) was processed by sodium dodecyl sulfate–polyacrylamide gel electrophoresis (SDS-PAGE) using NuPAGE Novex 4–12% Bis-Tris Mini Gels (Life Technologies). After electrophoresis, the slab gel was cut into strips and placed on chips. Then, peptides in the gel were electroblotted onto pre-wetted BLOTCHIP using an XCell II Blot Module (Life Technologies). The MALDI matrix, CHCA (Sigma-Aldrich, St. Louis, MO, USA), was applied onto the BLOTCHIP using an automatic matrix-dispensing machine (Protosera). All MS spectra were acquired on an UltraFlex II MALDI-TOF/TOF (Bruker Daltonics, Billerica, MA, USA) under previously defined conditions [28]. Each sample was measured in quadruplicate.

### MS/MS identification of K6F peptide on BLOTCHIP

Rat GEC medium supplemented with Kgp was subjected to SDS–PAGE and electroblotted onto BLOTCHIP as described above. External mass calibration was conducted on each prepared chip [28]. An MS/MS spectrum of K6F peptide was directly obtained from GCF samples blotted onto BLOTCHIP using the UltraFlex II instrument (Bruker Daltonics) in the LIFT mode. The fragmentation data were applied to a ”nonredundant” human database search (both NCBInr and Swiss-Prot) using the MASCOT MS/MS ion search program, version 2.1.0 (Matrix Science, Boston, MA, USA) interfaced with Biotools software (Bruker Daltonics).

### Peptide synthesis

All peptides were synthesized by ProteinPurify, Ltd. (Maebashi, Japan) at more than 95% purity, and were free of endotoxin contamination (SRL, Inc, Tokyo, Japan). The amino acid sequence of K6F was NH2-YEELQITAGRHGDDLRNTK-COOH (human and rat cytokeratin 6 residues 359–378), (MW = 2216.7) and that of the scrambled K6F (ScK6) was NH2-TKRNGRTALIHDGDQELYE-COOH (MW = 2216.7).

### Production of polyclonal antibodies against K6F and ScK6F

The K6F fragment and scrambled K6F peptide were used to raise anti-K6F and anti-ScK6F polyclonal antibodies, respectively. Antibodies were prepared according to a standard method [29].

### K6F antibody specificity

The specificity of the anti-K6F antibody was determined by western blot analysis. Rat GECs were treated with 10 nM Kgp for 1 h then lysed in lysis buffer (1% NP-40, 20 mM Tris–HCl, 150 mM NaCl) supplemented with 2 mM ethylenediamine tetra-acetic acid (EDTA), 2 mM ethyleneglycol-bis(β-aminoethyl ether)-N,N′-tetra-acetic acid, 1 mM phenylmethylsulfonyl fluoride, 4 mM sodium orthovanadate, and 400 mM sodium fluoride (Complete mini; Roche, Mannheim, Germany). The cell lysates were extracted by adding a twofold volume of sample buffer (100 mM Tris–HCl, 4% SDS, 2-mercaptoethanol, 20% glycerol), followed by sonication with a USP-300 sonicator (Shimadzu, Kyoto, Japan). Then, the extract was electrophoresed on a 12% SDS–polyacrylamide gel and probed for K6F expression with anti-K6F and anti-ScK6F antibodies (5 μg/ml). To determine the specificity of K6F antibody for the C-terminal region of cytokeratin 6 protein, K6F antibody was pre-incubated with 1 μg/ml of cytokeratin 6-C-terminal blocking peptide (GSSTIKYTTTS) (Sigma-Aldrich, St Louis, MO, USA) for 1 h. Full-length recombinant keratin 6B, KRT6B (AAH34535; amino acids 1–565) with a GST tag (MW = 87.7 kDa) was purchased from Abnova (Walnut, CA, USA).

### Immunoassay for K6F

This assay was performed according to a standard protocol for the competitive enzyme-linked immunosorbent assay (ELISA) system. In brief, microtiter plates (Nunc, Denmark) were precoated with 4 μg/ml K6F antigen in ELISA sample diluents (Neuromics, Edina, MN). After incubation at 4°C for 24 h, plates were washed (3×300 μl/well) with 0.2% Tween-20 in PBS (PBST) and blocked with 1% BSA and goat serum in PBS for 17 h at 4°C. Samples or standard peptides were pre-incubated with anti-K6F antibody (1 μg/ml) at 4°C overnight. The antibody/antigen complexes were then added to a well precoated with K6F peptide (100 μL/well). The plate was incubated at 37°C for 30 min then washed with PBST (300 μl/well) to remove excess antibody before addition of anti-rabbit IgG goat antibody conjugated to horseradish peroxidase (1:8000 dilution in ELISA sample diluents; 100 μl/well). Plates were then incubated at 37°C for 30 min, washed three times with PBST (300 μl/well) and any residual solution removed from the plate by flicking and slapping. Then, a solution of o-phenylenediamine in acetic acid (0.4 mg/ml) and hydrogen peroxide (0.06%) was added to the well (100 μl/well). After 6 min, the reaction was terminated by adding 50 μl stop solution (0.5 mol/l H_2_SO_4_) to each well. The absorbance of the solution was measured at 450 nm with a Multiskan Bichromatic Plate Reader (Labsystems, Helsinki, Finland) using the absorbance of the substrate solution as a blank. Background absorbance was obtained by putting a sample into uncoated wells. To determine the specificity of K6F ELISA assay, competitive inhibition of binding to the peptides raised against the N-terminal regions of cytokeratin 6 (6A, 6B or 6C) (Santa Cruz, Inc.), the C-terminal region (6F) and ScK6F peptide were used. Data obtained from standard curves displaying sensitivities ranging from 10 ug/ml down to 10 ng/ml.

### Immunohistochemistry

Human gingival tissues were obtained from five healthy volunteers (three male, two female; mean age = 46.7), and from five adult chronic periodontitis patients (three male, two female; mean age = 49.2) at Department of Periodontology, Kagoshima University, Kagoshima, Japan. Tissues were immunostained using anti-K6F antibody according to the method described in our previous study [23]. Half of the excised tissues were fixed in formalin, incubated in 20% sucrose solution, embedded in OCT and then stored at −80°C until use. The other half of the tissue samples were used to obtain membrane proteins. Staining of frozen tissue sections (4-μm thick) was carried out using the indirect immunoperoxidase diaminobenzidine (DAB) method with a DAKO LSAB+ System HRP kit (KO679; DakoCytomation, Carpinteria, CA, USA).

### Immunofluorescence staining

Rat GECs were cultured on poly-D-lysine coated chamber slides (Lab-Tek, Nunc, Denmark). Immunofluorescence staining of cells was performed after treatment with 50 nM Kgp for 6 h. Briefly, cells were washed with PBS, fixed with cold methanol and permeabilized with 1% Triton X-100, followed by a treatment with blocking buffer (0.1% Triton X-100 containing 1% BSA and normal goat serum). Sections were incubated at room temperature for 1 h with anti-cytokeratin 6 C-terminal region monoclonal antibody (Clone LHK6B) (IMGENEX, San Diego, CA, USA; 1:100 dilution), anti-ScK6F rabbit polyclonal antibody or isotype-matched control IgG. Slides were washed with PBS and incubated with AlexaFluor 488-conjugated goat anti-mouse IgG (1:500 dilution; Molecular Probes) or AlexaFluor 594-conjugated donkey anti-rabbit IgG (1:500 dilution; Molecular Probes) secondary antibody. After washing, nuclei were stained with DAPI (1:500 dilution; Nakalai Tesque, Kyoto, Japan). Concanavalin A (ConA)-conjugated AlexaFluor 594 (1:100 dilution; Invitrogen) was used as a cell surface localization marker. Samples were observed with a fluorescence microscope (Axioskop; Carl Zeiss, NY) with an excitation filter range of 470–490 nm and an emission filter at 580 nm. Five digitized images (×400 magnification) were captured at random for each case. Fluorescence data were exported from generated images and were expressed as a percentage of fluorescence intensity relative to that of control samples as described previously [30]. For K6F localization on cultured HGFs, cells were grown under the same conditions (except for an additional incubation with 1 μg/ml fluorescein isothiocyanate (FITC)-labeled K6F or ScK6F antibody) and co-stained with ConA-conjugated AlexaFluor 594 for 30 mins, then fixed with 1.5% paraformaldehyde in PBS without permeabilization.

### Flow cytometric analysis

Rat GEC monolayers incubated with Kgp or TCLK-treated Kgp were gently dispersed and resuspended at a final concentration of 3×10^6^ cells/ml. After washing with PBS, cells were fixed with OptilyseC (Becton Dickinson, Franklin Lakes, NJ) containing 0.2% Triton X-100 (Sigma-Aldrich). Next, cells were washed with 0.2% Triton X-100 in PBS (washing buffer) and incubated with anti-cytokeratin-6 antibody (20 μg/ml) or with the same concentration of non-immune serum for 1 h in washing buffer, followed by incubation with FITC-conjugated secondary antibody (ICN Pharmaceuticals, Aurora, OH) for 30 min. Fluorescence was analyzed with a FAC-Scan analyzer (Beckman Coulter, Fullerton, CA).

### Western blot analysis

Rat GEC suspension (5×10^6^ cells) was seeded into 100-mm cell culture dishes and treated with Kgp (10 nM), RgpB (5 or 10 nM) for 1, 6 and 12 h or ultrapure *P*. *gingivalis* LPS or *A*.*a* LPS (0.1, 1 or 10 μg/ml) (Invitrogen) for 6 h. Cells were then separated into cytoskeletal and cytosolic fractions using a cell compartment kit (Qproteome; Qiagen, Valencia, CA) according to the manufacturer’s instructions, except that a serine protease inhibitor (diisopropyl fluorophosphate (10 mM)) was added to the extraction buffer. Cytoskeletal fractions were washed twice with Ca^2+^/Mg^2+^-free PBS and treated with extraction buffer. Protein concentration was measured using the Bradford protein assay (Bio-Rad Laboratories, Hercules, CA) using BSA as a standard according to the manufacturer’s protocol. Protein extracts were analyzed by SDS-PAGE (10 μg/lane) using a 12% gel and transferred from the gel onto polyvinylidene fluoride membranes to perform immunoblotting analysis using anti-cytokeratin 6 monoclonal antibodies (1:300) or anti-vimentin monoclonal antibodies (1:500; both from Santa Cruz Biotechnology, Santa Cruz, CA,USA), followed by a goat secondary antibody against mouse IgG (ICN Pharmaceuticals, Aurora, OH, USA). Finally, the membrane was developed using an ECL kit (Amersham Pharmacia Biotech, Piscataway, NJ, USA). The density of cytokeratin 6 bands was measured using NIH Image v.1.61 software.

### Mitogen-Activated Protein Kinase (MAPK) and Akt activation assay

Protein expression was analyzed as described previously [25], with modifications. Briefly, HGF (6×10^4^ cells) were seeded onto 60-mm cell culture dishes. After stimulation for 15, 30 and 60 min with K6F at 1 μg/ml, cells were washed twice with Ca^2+^/Mg^2+^-free PBS and lysed with the same lysis buffer used for GEC lysis (see above). From this cell lysate solution, 20 μg of protein was subjected to SDS-PAGE, transferred onto polyvinylidene fluoride membranes and probed for MAPK and Akt activation by immunoblotting using MAPK assay kits (containing polyclonal antibodies against phospho-p38, phospho-Jun N-terminal kinase (JNK)/stress-activated protein kinase and phospho-p44/42) and anti–phospho-Akt antibody (Cell Signaling Technology Inc., Beverly, MA, USA).

### Invasion assay

HGF migration was assessed using Matrigel chambers (two wells separated by Matrigel on a 6.5-mm diameter polycarbonate membrane with 8-μm pores; Corning Costar, MA) as described previously [31] with modifications. Cells were starved overnight in serum-free medium containing 0.1% BSA and then pre-incubated with SB203580 (a p38 inhibitor), SP600125 (a JNK inhibitor) or U0126 (a p42/p44 inhibitor) for 1 h before stimulation with K6F. The lower compartment of the well received 500 μl of control medium supplemented with K6F (1 μg/mL), ScK6F (1 μg/mL), plain medium as a negative control, or platelet-derived growth factor (10 ng/ml) as a positive control. The upper compartments received 200 μl of cells (25,000) in serum-free medium containing 0.1% BSA. The cells were incubated for 12 or 24 h at 37°C. Cells on the upper surface of the membrane were completely removed by sweeping with cotton swabs and cells on the lower surface of the membrane were fixed in methanol and stained with hematoxylin. Five digitized images (×40 magnification) from the migrated cell area were captured at random and cells were counted. Migratory activity was expressed as the relative invasion capacity compared with that measured in control medium.

### Measurement of cytokines

HGFs were seeded in 96-well flat-bottomed culture plates at 1×10^5^ cells per well and grown to confluence (mean: 2×10^5^ cells per well) before addition of 1 μg/ml K6F or ScK6F. MCP-1, IL-8, and IL-6 in the cell supernatants were measured by ELISA assay kits according to the manufacturer’s instructions (R&D Systems, MN). To determine the involvement of MAPKs in the release of these cytokines, cells were pre-incubated with various concentrations of MAPK inhibitors for 1 h before stimulation with K6F or ScK6F.

### Cell viability assay

Cell viability was determined by the methylthiazolyl-diphenyl-tetrazolium bromide (MTT) assay as described previously [32]. Briefly, after stimulation of cells with various concentrations of peptides (1–25 μg/ml) in six-well plates for 48 h, MTT solution was added to each well. Three hours later, dimethyl sulfoxide was added and the absorbance of the solution was measured at 570 nm with an automatic microplate reader (ImmunoMini NJ-2300; InterMed, Tokyo, Japan). The percentage survival of cells was calculated using DMSO-treated cells as a standard.

### Statistical analysis

All data are expressed as the mean ± SD. Differences between groups were assessed by analysis of variance (ANOVA) followed by Dunnett’s multiple comparison test (SPSS Inc., Chicago, IL, USA). Statistical analyses of MS data were conducted using ClinPro Tools version 2.2 (Bruker Daltonics) as previously described[28]. *P* < 0.05 was considered statistically significant.

## Results

### Proteodegradome of K6F in GCF from PD patients and in rat GEC supernatant

**Fig 1:**
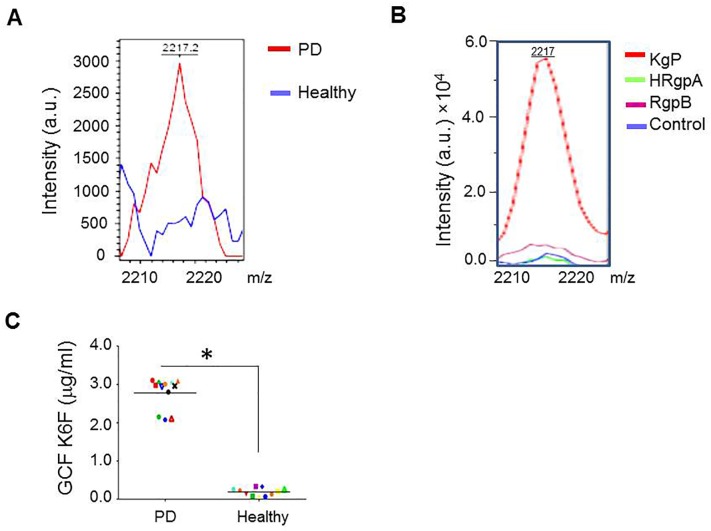
MALDI-TOF mass spectrometry profiles of GCF and rat GEC culture supernatants and K6F concentrations in GCF from PD patients and healthy volunteers. A: The proteins in GCF were extracted from the strips into the assay buffer and analyzed by MALDI-TOF mass spectrometry in arbitrary units (a.u.). The profile of GCF from PD patients on K6F peptides (red line) was superimposed onto that of healthy subjects (blue line). B: Profile of K6F detected in rat GEC supernatants. Activated gingipains were incubated with primary cultures of rat GECs, and supernatants were collected. A peak of K6F was detected in supernatants from rat GECs treated with Kgp (red line) at a molecular weight of 2217. C: GCF samples were collected and K6F titers were quantitatively measured by ELISA. Chart shows a dot plot of K6F levels in individual patients. Experiments were performed two times in triplicate. *P<0.001 versus PD.

GCF is an inflammatory exudate that can be collected at the gingival margin or within the gingival crevice [33] and is available for assessment in the active phase of PD. GCF samples from PD patients and healthy control individuals were subjected to BLOTCHIP-MS analysis. In the GCF of PD patients, a major peak was detected that had a molecular weight of 2217.2 and was of significantly higher magnitude than the same peak in GCF from subjects with no periodontal disease (Fig. 1A). As described previously [34], subjects suffering from destructive periodontitis have antibodies specific for gingipains in both serum and GCF. We next identified this peptide in the media from rat GECs cultured in the presence of Kgp, HRgpA or RgpB. The mass spectrum of medium supplemented with Kgp contained two major peaks, one at *m/z* 2217 (referred to as K6F) (Fig. 1B) and one at *m/z* 2230 (data not shown). The amount of K6F measured in media from rat GECs cultured with Kgp was much higher than that in cells cultured with RgpA or RgpB, which were little more than the control level. Amino acid sequencing of K6F revealed a 19 amino acid residue sequence (YEELQITAGRHGDDLRNTK) corresponding to a peptide comprising amino acids 359–378 of human cytokeratin 6B (the full length of which is 564 residues) [35]. The cleavage sites at Lys_357_-Tyr_358_ and Lys_378_-Gln_379_ were consistent with the substrate specificity of Kgp, which cleaves peptide bonds strictly at the carboxy-terminal side of Lys residues [36]. To measure K6F in patient samples, we prepared K6F antibody and analyzed K6F levels in GCF samples by ELISA. K6F levels in the GCF from PD patients (2.78 ± 0.4 μg/ml) were much higher than in that from healthy samples (0.19 ± 0.1 μg/ml) (*P* < 0.001; Fig. 1C). K6F was not cytotoxic to cultured rat GECs (data not shown). To test the specificity of the antibodies used against these peptides, we performed western blot analyses using anti-K6F antibody and anti-scrambled KF6 peptide (ScK6F) antibody (S1 Fig.). After treatment with Kgp, some smaller molecules were observed at ~27 kDa, in addition to cytokeratin-6 (full-length) at 57 kDa (left panel). The K6F-positive band could be removed by competitive inhibition using the cytokeratin-6-C-terminal blocking peptide, indicating the specificity of anti-K6F antibody to the cytokeratin-6-C-terminal region associated with the Kgp cleavage sites. The antibody against ScK6F recognized only its cognate antigen (right panel). In addition, the K6F ELISA showed no significant cross-reactivity with human N-terminal regions of cytokeratin 6 the C-terminal region (6F) and ScK6F, indicating that the K6F ELISA recognized a neoepitope from human and rat cytokeratin 6 amino acid 359–378 (data not shown).

### K6F Localization in human gingival tissues

**Fig 2:**
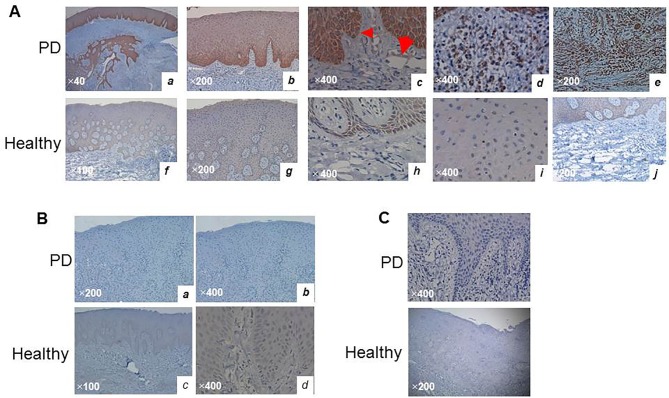
Immunohistochemistry of K6F in human gingival tissues. Tissues from PD and healthy volunteers were snap-frozen and incubated with polyclonal antibody against (A) K6F or (B) ScK6F (5 μg/ml). C: Negative immunostaining for K6F in healthy and PD patients by isotype-matched control IgG. Arrow and arrowhead in Panel A(c) indicate K6F immunoreactivity on endothelial cells and the basal cell layer, respectively. Data are representative of three independent experiments. Scale bar, 50 micron.

Localization of the K6F antigen in the gingival tissues of PD patients and healthy individuals was investigated using the K6F-specific antibody. K6F was detected in all samples from PD patients and was present in the gingival epithelia from the basal layer (Fig. 2A, C, arrowhead) to the spindle cell layers with reducing density (a and b). There was also scattered K6F in capillary endothelial cells (c, arrow), infiltrating inflammatory cells, and fibroblast-like cells (d and e) in the connective tissue. In healthy volunteers, there was no K6F in the connective tissue layers but faintly positive in the epithelia (Fig. 2A, F–J). No tissues or cells in samples from PD patients or healthy volunteers were stained with anti-ScK6 antibodies (Fig. 2B) or isotype-matched control IgG (Fig. 2C). These results suggest that K6F antigen increases in PD tissues, possibly due to the action of Kgp, but not in healthy periodontal tissues [7].

### Effect of Kgp on cytokeratin 6 levels in the cytoskeletal fraction and supernatant of rat GEC cultures

**Fig 3:**
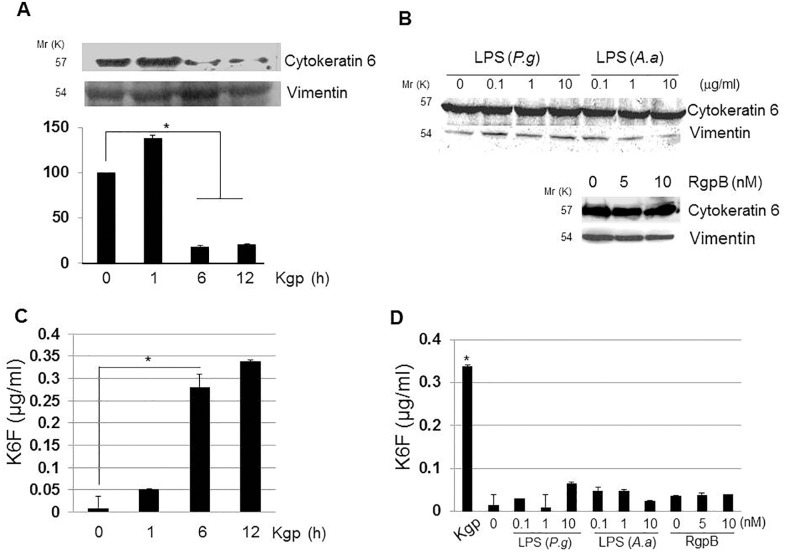
Effects of Kgp on cytokeratin-6 in the cytoskeleton fraction and K6F in rat GEC culture supernatants. A: Cytoskeletal protein was extracted from rat GECs treated with 10 nM Kgp, and cytokeratin-6 levels were measured by immunoblotting. Bands were quantified by densitometry. B: Cells were treated with *P*. *gingivalis* (*P*.*g*), *A*. *actinomycetemcomitans* (*A*.*a*) LPS or RgpB for 6 h before measurement of cytokeratin-6 levels in the cytoskeleton fraction. Vimentin was used as a loading control. Data are representative of three independent experiments. C: K6F in rat GEC supernatant after incubation with 10 nM Kgp as measured by ELISA. D: *P*.*g*., *A*.*a*. LPS or RgpB were used instead of Kgp. Data are the mean ± SD (n = 3).*P<0.05.

Western blotting of the rat GEC cytoskeleton fraction, using vimentin as a loading control, revealed that Kgp degraded cytokeratin 6 in a time-dependent manner between 6 and 12 h (Fig. 3A). Densitometric evaluation of the bands indicated an increase in cytokeratin 6 after 1 h treatment of rat GECs with Kgp, and a significant decrease after 6–12 h treatment with Kgp (*P < 0*.*01*). Cytokeratin 6 in the cytoskeletal fraction did not change after incubation of rat GECs with *P*. *gingivalis* lipopolysaccharide (LPS), *A*. *actinomycetemcomitans* LPS, or RgpB (Fig. 3B), indicating that the cytokeratin 6 change was caused by Kgp. Kgp increased K6F significantly (*P* < 0.05) in the rat GEC culture supernatant in a time-dependent manner from 6 h after treatment (Fig. 3C), whereas RgpB, *P*. *gingivalis* LPS and *A*. *actinomycetemcomitans* LPS did not increase K6F levels (Fig. 3D). This result suggests that Kgp can cleave K6F from cytokeratin 6 in rat GECs.

### Cytokeratin-6 degradation in rat GECs by Kgp treatment

**Fig 4:**
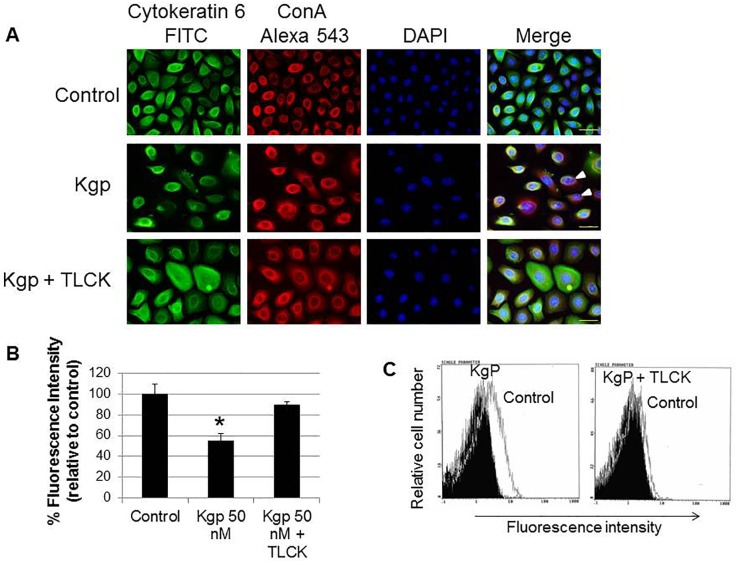
Rat GEC cytokeratin-6 degradation by Kgp. A: Cells were either untreated (control, top), treated with 50 nM Kgp for 6 h or treated with Kgp inactivated by TLCK. After treatment, cells were stained with anti-cytokeratin 6 antibody (FITC, green) and Concanavalin A (ConA-Alexa 543, red), followed by DNA staining with DAPI (blue). Images were obtained by a fluorescence microscope at 400× magnification. Arrowhead indicates degraded cytokeratin-6 protein in rat GECs. Scale bar = 30 μm. B: Images were captured and fluorescence intensity was quantitated using Image J software. The percentage fluorescence intensity relative to control is shown and expressed as mean ± SD (n = 3). C: Flow cytometry of cytokeratin-6 protein is shown as the geometric mean of the fluorescence intensity (x-axis). The shaded histograms denote fluorescence in Kgp-treated cells before the assay. Data are representative of three independent experiments. *P<0.05.

Next, to confirm Kgp cleavage of cytoplasmic cytokeratin 6, rat GECs were analyzed by immunofluorescence staining using human cytokeratin 6 C-terminus-specific antibody (green) and concanavalin A (Con A; red) that binds to cell surface carbohydrates. DNA staining with DAPI (blue) was used to define nuclei. In untreated rat GECs (top), cytokeratin 6 formed a dense mesh of filaments throughout the cytoplasm and was also coincident with ConA-bound cell surface carbohydrates (indicated by yellow fluorescence; Fig. 4A). After treatment with Kgp (middle), the distribution of cytokeratin 6 changed to fine reticular networks and the filaments were weakly or non-stained in most rat GECs. The merged image shows decreased yellow fluorescence (arrowheads), indicating the loss of cytokeratin 6 antigenicity, which likely occurs owing to degradation of the protein by Kgp. Inhibition of Kgp activity with TLCK significantly blocked cytokeratin 6 degradation (bottom). Neither anti-ScK6F antibodies (S2 Fig.) nor an isotype-matched control IgG (S3 Fig.) recognized rat GECs. Kgp significantly reduced cytokeratin 6 fluorescence to about 50% compared with the control cells (*P < 0*.*05*), and the Kgp inhibitor blocked this reduction of fluorescence (Fig. 4B). Cytokeratin 6 reduction by Kgp was also seen by flow cytometry (Fig. 4C). These results confirmed that Kgp can degrade cytokeratin-6 in rat GECs.

### K6F localization on HGFs

**Fig 5:**
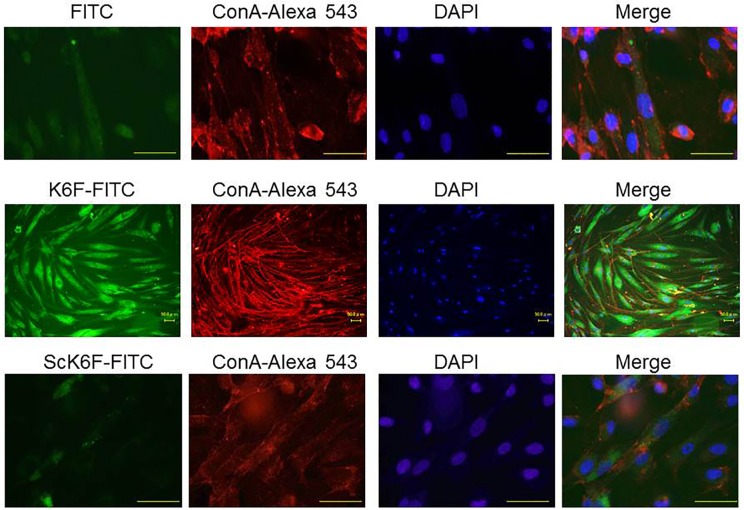
Presence of K6F on human gingival fibroblasts. Cells were either untreated (control, top), or treated with FITC (green)-labeled K6F (middle) or FITC-labeled ScK6F antibody (bottom) (1 μg/ml). Cells were then counterstained with Alexa 543-labeled-ConA (red) and DAPI (blue). All images were obtained under an immunofluorescence microscope. Scale bar = 50 μm. Data are representative of three independent experiments.

Fibroblasts in the connective tissues are responsible for sustaining inflammatory responses in PD [37]. To screen for K6F localization in HGFs, cells were analyzed by immunofluorescence staining using FITC-conjugated to K6F or ScK6F antibody (green) and ConA (red), followed by DNA staining with DAPI (blue). K6F was observed within 30 min after the start of incubation (Fig. 5). Faint red ConA fluorescence was observed along the plasma membrane in cells treated with FITC alone (top). In cells treated with FITC-conjugated K6F antibody (middle), dense green fluorescence was distributed throughout the cell membrane, whereas in most HGFs incubated with the FITC-conjugated ScK6F antibody there was only extremely faint green fluorescence (bottom). Thus, K6F is localized on the surface of HGFs. None of the FITC-conjugated antibodies were cytotoxic to HGFs (data not shown).

### Role of p38 and JNK1/2 in K6F-induced cytokine release from HGFs

**Fig 6:**
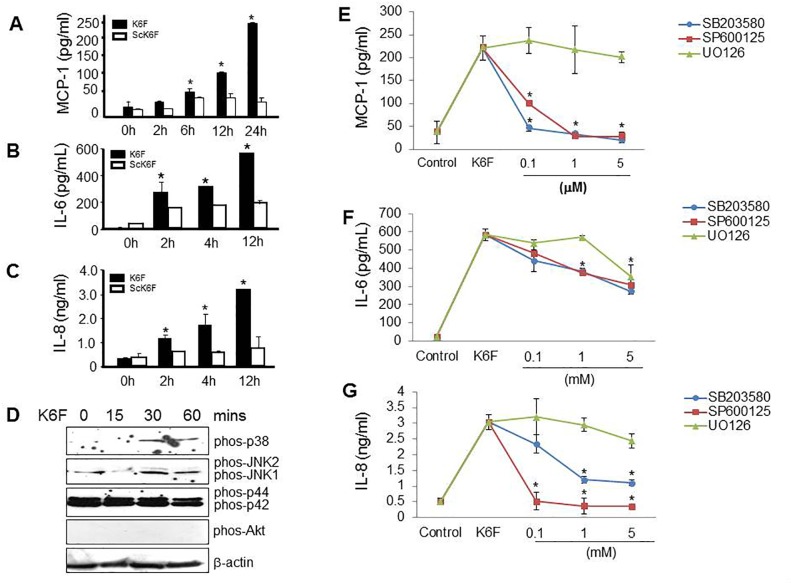
K6F-induced cytokine release from HGFs. Cells were incubated with 1 μg/mL K6F or ScK6F before ELISA measurement of the levels of (A) MCP-1, (B) IL-6 and (C) IL-8 in the culture medium. D: Cells were incubated with 1 μg/mL K6F for 0–60 min, and the activation of p38 MAPK, JNK1/2, p42/p44 and Akt was determined by western blot analysis using antibodies that specifically recognize the activated forms of these kinases. Cells were pretreated with SB203580 (SB; a p38 inhibitor), SP600125 (SP; a JNK inhibitor) or U0126 (UO; a p42/p44 inhibitor) for 1 h, then incubated with K6F before measuring the levels of (E) MCP-1, (F) IL-6 and (G) IL-8in the culture medium by ELISA. Values denote mean ± SD (n = 3). *P<0.05 *vs*. control.

A wide variety of cytokines, chemokines and their receptors are synthesized by gingival fibroblasts, epithelial cells, endothelial cells and inflammatory cells in PD [38]. To investigate whether K6F acts as an inducer of these PD-related cytokines, the MCP-1, IL-6 and IL-8 content of culture supernatants from HGFs treated with K6F or ScK6F was measured by ELISA. The addition of K6F at a dose seen in clinical samples increased MCP-1 (Fig. 6A), IL-6 (Fig. 6B) and IL-8 (Fig. 6C) secretion in a time-dependent manner (*P* < 0.05). These mediators did not increase significantly following ScK6F treatment (Fig. 6A–C). To further study the mechanism of this action of K6F, we examined the activity of the MAPK (p38, JNK1/2, p42/p44) and Akt pathways by western blot analysis using antibodies against the phosphorylated (active) forms of these proteins. Phosphorylated p38 MAPK and JNK1/2 increased following K6F treatment, but the level of phosphorylated p42/p44 and Akt did not change (Fig. 6D). p38 and JNK1/2 phosphorylation was detected at 30 min after exposure to K6F and was sustained until 60 min. This result indicates that K6F stimulates HGFs *via* the activation of the p38 and JNK1/2 pathways.

To further demonstrate that the K6F-elicited cytokine release was dependent on MAPK signaling, HGFs were pre-incubated with SB203580 (a p38 inhibitor), SP600125 (a JNK inhibitor) or U0126 (a p44/p42 inhibitor) before stimulation with K6F. SB203580 and SP600125, but not U0126, significantly attenuated secretion of MCP-1 (Fig. 6E), IL-6 (Fig. 6F), and IL-8 (Fig. 6G) in a dose-dependent manner. These data confirm that K6F induces cytokine secretion from HGFs *via* activation of the p38 and JNK1/2 pathways.

### Dependence of K6F-induced HGF migration on activation of p38 and JNK1/2

**Fig 7:**
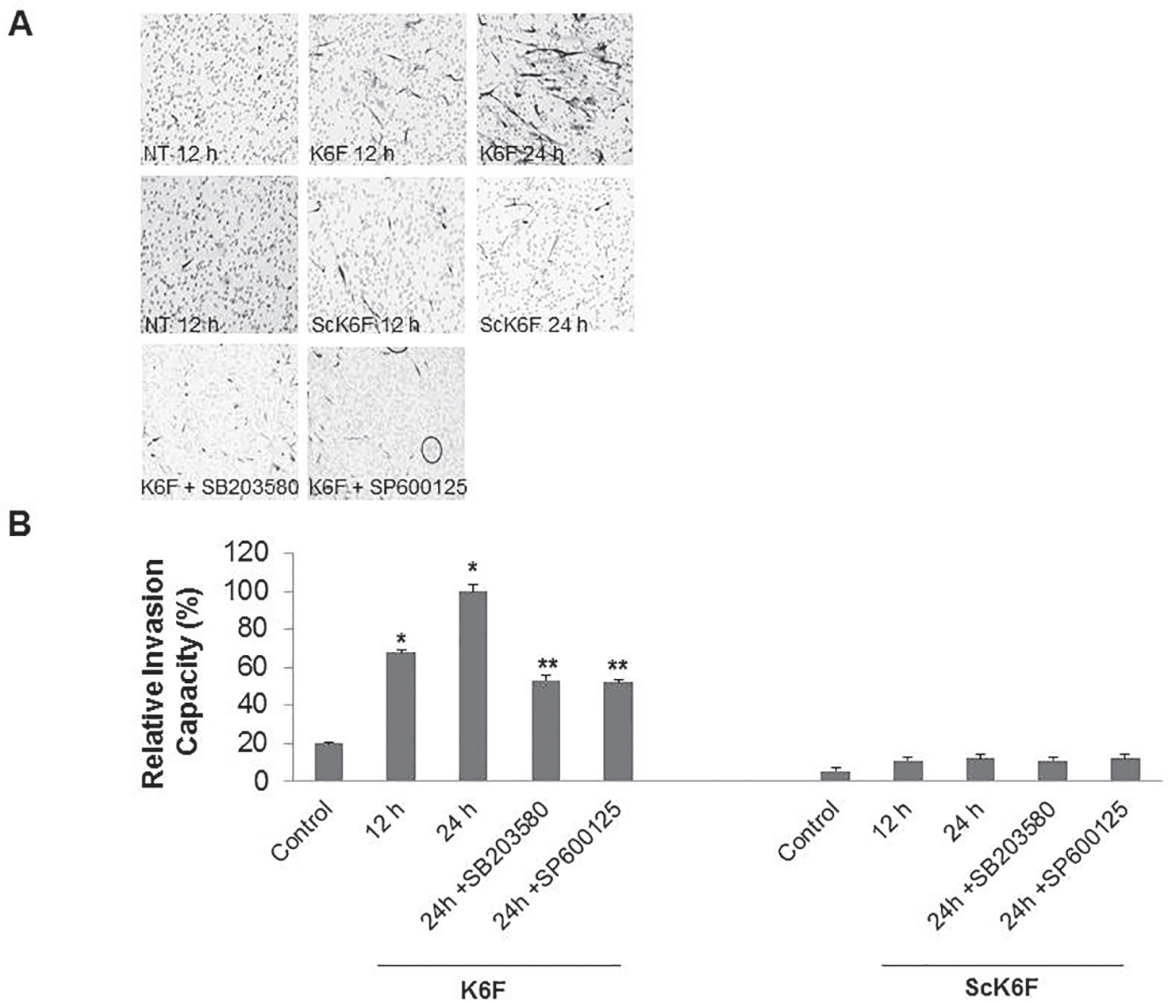
K6F-induced HGF migration and its inhibition by p38 MAPK and JNK1/2 inhibitors. A: Cells were seeded into the upper chamber of a transwell apparatus equipped with a 6.5-mm polycarbonate filter (8-μm pore) layered with 40 μg of Matrigel. Lower wells contained 500 μl of control medium supplemented with 1 μg/ml of K6F or ScK6F. The Matrigel chambers were incubated for 12 or 24 h in the presence or absence of 1 μM SB203580 or 1 μM SP600125 in the upper chamber. Cells in the top well were completely removed and cells on the underside of the membrane were counterstained with Mayer’s hematoxylin. B: Images were captured and cells were counted at ×40 magnification. The migration-enhancing effect is shown as the relative invasion capacity of the number of migratory cells in the sample versus that in control media. Data are the mean ± SD (n = 3) of two independent experiments. ‘NT’ denotes no treatment. *P<0.05 *vs*. control. **P<0.05 *vs*. K6F alone.

We also investigated the mechanism of the K6F effect on HGF migration in the Matrigel assay. HGFs underwent shape change from polygonal to spindle fibroblast-like morphology in the presence of K6F. This K6F effect was inhibited by both SB203580 and SP600125 (Fig. 7A). K6F markedly enhanced HGF migration (by 3.38- and 5.0-fold after incubation for 12 and 24 h, respectively) whereas ScK6F had no significant effect on cell migration (Fig. 7B). The K6F-mediated increase in HGF migration at 24 h was reduced to just 2.6-fold by the presence of either SB203580 or SP600125 (Fig. 7B). In contrast, U0126 had no effect on this activity (data not shown).

## Discussion

We identified a short peptide (K6F) as a novel Kgp cleavage product of human cytokeratin 6, high levels of which were found in the GCF of PD patients. Kgp degradation of cytokeratin-6 on the surface of rat GECs led to the release of K6F, and we showed a possible mechanism by which K6F elicits gingival inflammation.

Epithelial cells maintain their structure mostly through the support of keratins and the extracellular matrix. Survival and propagation of oral epithelial cells depend on stable cytoskeleton arrangement, as do normal physiological processes and tissue homeostasis [12]. Previous studies reported that breakdown of the extracellular matrix [39] and microfilaments [40] by *P*. *gingivalis* enhances the chronic tissue destruction seen in PD. Kgp is stable and active in the presence of reducing agents at or above pH 8.0, which is the pH of the GCF of patients with PD [41], and is thus likely to be the most active protease in the GCF of PD patients [42]. Various bacterial and viral proteases cleave host cell keratins [43–45] and a recent study demonstrated soluble cytokeratin fragments in the serum and urine [46]. Delayed cell proliferation, short cell survival, and induction of the inflammatory network occur following keratin-6 deletion [18,47] or keratin-6 mutations [19,20]. Our data showing significantly increased levels of K6F in the GCF of PD patients (Fig. 1) and Kgp cleaves cytokeratin-6 in host rat GECs, releasing K6F into the extracellular milieu (Figs. 3–6) may suggest that cytokeratin-6 degradation by Kgp is a contributory factor in rat GEC cytoskeleton collapse, leading to cell changes and inflammation similar to that seen in periodontitis tissues.

Using gingipains, *P*. *gingivalis* can migrate across the basement membrane and reach the underlying connective tissue [48]. Important features of the gingipains involved in PD (Rgp and Kgp) include the ability to attach to and invade host cells, disseminate within host tissues, and subvert host immunological surveillance and defense mechanisms [49]. Proteolytic activity of Kgp but not Rgp was reported to act as a positive inducer of periodontal bone loss *in vitro* and *in vivo* by degrading osteoprotegerin [50].Kgp can directly hydrolyze components of the extracellular matrix such as fibronectin [51] and shed syndecan-1 from GECs [52]. However, the mechanisms by which Kgp disrupts host cell intermediate filaments and induces periodontal inflammation are unknown. We revealed for the first time that GEC disruption through cytokeratin-6 cleavage by Kgp produces K6F, which elicits cytokine secretion from HGFs and enhances HGF migration, both of which are associated with the pathophysiology of PD.

Kgp at concentrations of 50–100 nM enhances osteoclast formation by degrading osteoprotegerin [53], which is approximately the Kgp concentration detected in GCF from adult PD patients [54]. Kinane et al. [40]reported that Kgp degrades actin at a maximum concentration of 3 μg/ml (50 nM), the same concentration used to treat rat GECs in the present study. Accordingly, it is likely that cytokeratin-6 degradation by Kgp and the subsequent release of K6F can occur *in vivo*.

A wide variety of type I and II keratins and their fragments were recently detected in the GCF of PD patients using proteomic based liquid chromatography-electrospray ionization/multi-stage mass spectrometry (LC-ESI/MS/MS), but K6F was not identified [55]. Using the BLOTCHIP-MS method [28], we identified K6F. Our success in K6F detection might be due to differences in the MS technique employed. The BLOTCHIP-MS method enables direct electric transfer of peptides from the 1-D PAGE gel to the target plate. Electrophoresis using this system removes high-molecular-weight proteins that hinder the MS of peptides, resulting in high reproducibility for peptide quantitation in GCF and cellular supernatants [27]. This advantage may facilitate biomarker discovery and further understanding of PD pathophysiology leading to the development of targeted PD therapies.

HGFs are responsible for the formation and turnover of the extracellular matrix [56]. As PD enhances the apical migration of junctional epithelial cells, gingival fibroblasts invade the surrounding connective tissue [57,58]. Increased secretion of various pro-inflammatory cytokines occurs in periodontal tissues. MAPKs are involved in cytokine release, cytoskeletal reorganization [59] and cell migration [60]. The phosphoinositide 3-kinase (PI3K)/Akt signaling pathway regulates cell proliferation, apoptosis, and cell migration [61]. Both pathways are activated in PD tissues [62,63]. Our findings that p38 MAPK and JNK1/2, but not Akt, are phosphorylated by K6F stimulation likely explains the ability of K6F to induce the release of IL-6, IL-8 and MCP-1 and to enhance HGF invasion (Fig. 7). Compared with healthy subjects, the gingival tissues and GCF of patients with chronic PD are reported to have significantly increased amounts of proinflammatory cytokines such as IL-6, IL-8 and macrophage chemoattractant protein 1 (MCP-1) [64,65]. These cytokines may recruit mononuclear cells, consisting mainly of T cells and macrophages [66], into PD lesions. Moreover, K6F-elicited MCP-1 release from HGFs suggests their participation in monocyte recruitment in gingival tissues of adult PD patients. We demonstrated in our immunofluorescence studies that K6F was present on HGFs (Fig. 5). Proteolytic release of K6F by Kgp may alter cell membrane fluidity, and induce cellular signaling entry to the host cells [67,68]. The identification of a K6F receptor on HGFs would confirm the existence of a K6F-mediated pathway of cell activation and provide further understanding of gingival tissue damage by inflammation in PD. However, such a receptor has yet to be found. To our knowledge, this is the first report to show the involvement of the *P*. *gingivalis*-derived protease Kgp in the cleavage of cellular intermediate filaments of the cell cytoskeleton. The possible correlation between the severity of PD and the K6F levels in the GCF and serum of PD patients is under investigation, and could lead to the development of an ELISA-based assay to measure K6F as a biomarker of PD.

## Supporting Information

S1 Fig Specificity of anti-K6F antibody to the C-terminal region of cytokeratin 6. To investigate the specificity of the K6F antibody to the C-terminal region of cytokeratin 6, cell lysates of rat GECs treated with Kgp were analyzed by SDS-PAGE, followed by immunoblotting. GST-tagged full-length recombinant cytokeratin-6 (Rec-K6) expressed in *E*. *coli* had a mass of 87.7 kDa. A: Cell lysates of rat GECs treated with Kgp and Rec-K6. Left, anti-K6F antibody; Right, anti-K6F antibody pre-incubated with K6-C-terminal blocking peptide. B: Cell lysates and ScK6F peptide were probed with anti-ScK6F antibody. K6F antibody is specific to a C-terminal region of cytokeratin 6, and no cross-reactivity of ScK6F against K6F was observed. The mass (in kDa) of protein standards is indicated in the left lane.
(TIF)

S2 Fig No reactivity of anti-ScK6F antibody to rat GECs by double fluorescence staining analysis. Rat GECs were incubated in the presence or absence of 50 nM Kgp for 6 h, double-stained using anti-ScK6F antibody (FITC, green) and ConA (Alexa543, red), and counter-stained for DNA with DAPI (blue). All images were obtained with a fluorescence microscope at ×400 magnification. Scale bar = 30 μm.
(TIF)

S3 Fig No reactivity of control rabbit IgG to rat GECs by double fluorescence analysis. Rat GECs were incubated in the presence or absence of 50 nM Kgp for 6 h, double-stained using control rabbit IgG (FITC, green) and ConA (Alexa543, red), and counter-stained for DNA with DAPI (blue). All images were obtained with a fluorescence microscope at ×400 magnification. Scale bar = 30 μm.
(TIF)
