# Supplementary figures and images for: Cleavage of Host Cytokeratin-6 by Lysine-Specific Gingipain Induces Gingival Inflammation in Periodontitis Patients
Source: PLoS One. 2015 Feb 17;10(2):e0117775. doi: 10.1371/journal.pone.0117775 (PMC4331500; doi:10.1371/journal.pone.0117775)

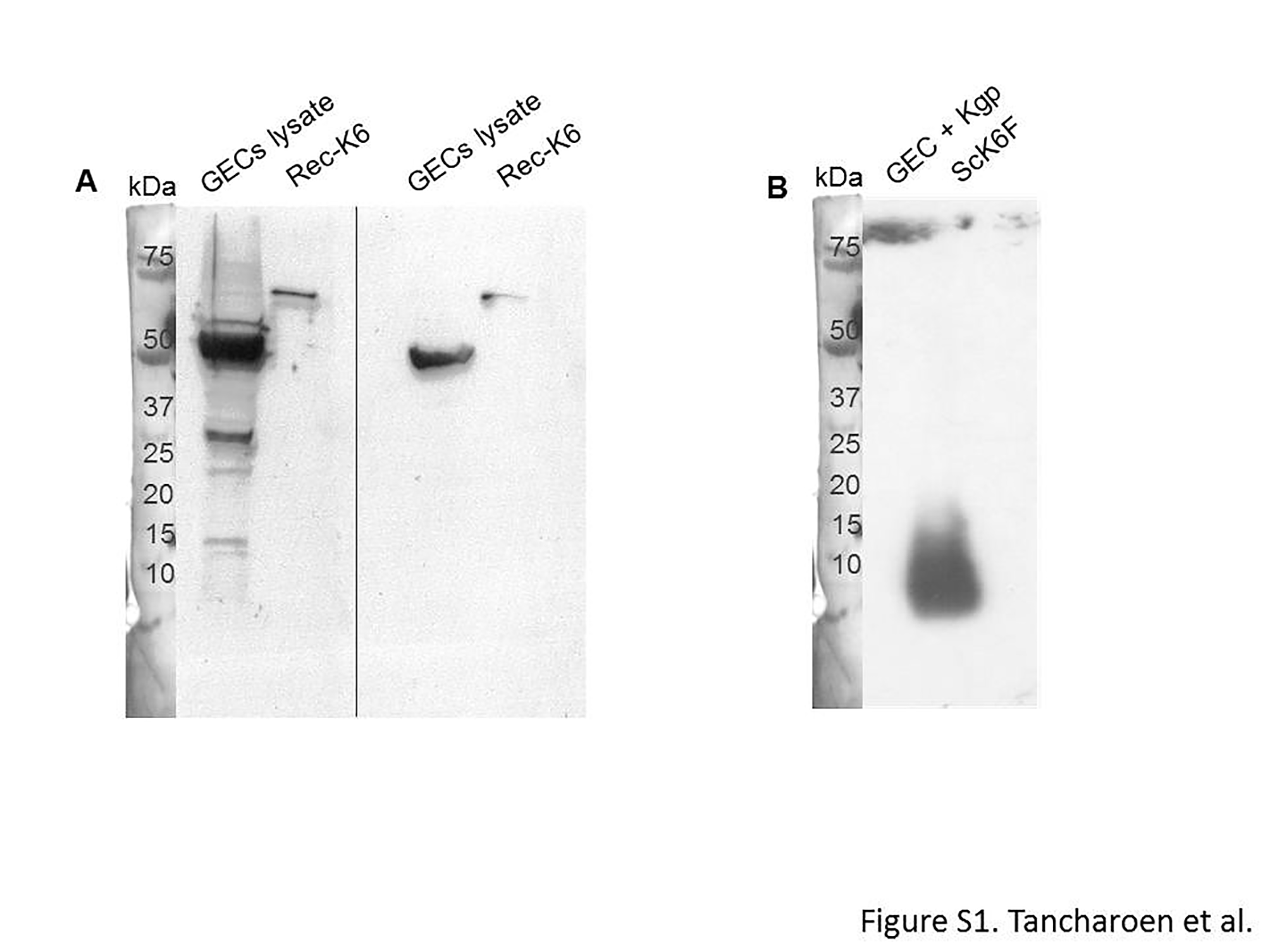

Supplement: S1 Fig — To investigate the specificity of the K6F antibody to the C-terminal region of cytokeratin 6, cell lysates of rat GECs treated with Kgp were analyzed by SDS-PAGE, followed by immunoblotting. GST-tagged full-length recombinant cytokeratin-6 (Rec-K6) expressed in E. coli had a mass of 87.7 kDa. A: Cell lysates of rat GECs treated with Kgp and Rec-K6. Left, anti-K6F antibody; Right, anti-K6F antibody pre-incubated with K6-C-terminal blocking peptide. B: Cell lysates and ScK6F peptide were probed with anti-ScK6F antibody. K6F antibody is specific to a C-terminal region of cytokeratin 6, and no cross-reactivity of ScK6F against K6F was observed. The mass (in kDa) of protein standards is indicated in the left lane. (TIF) [file pone.0117775.s001.tif]

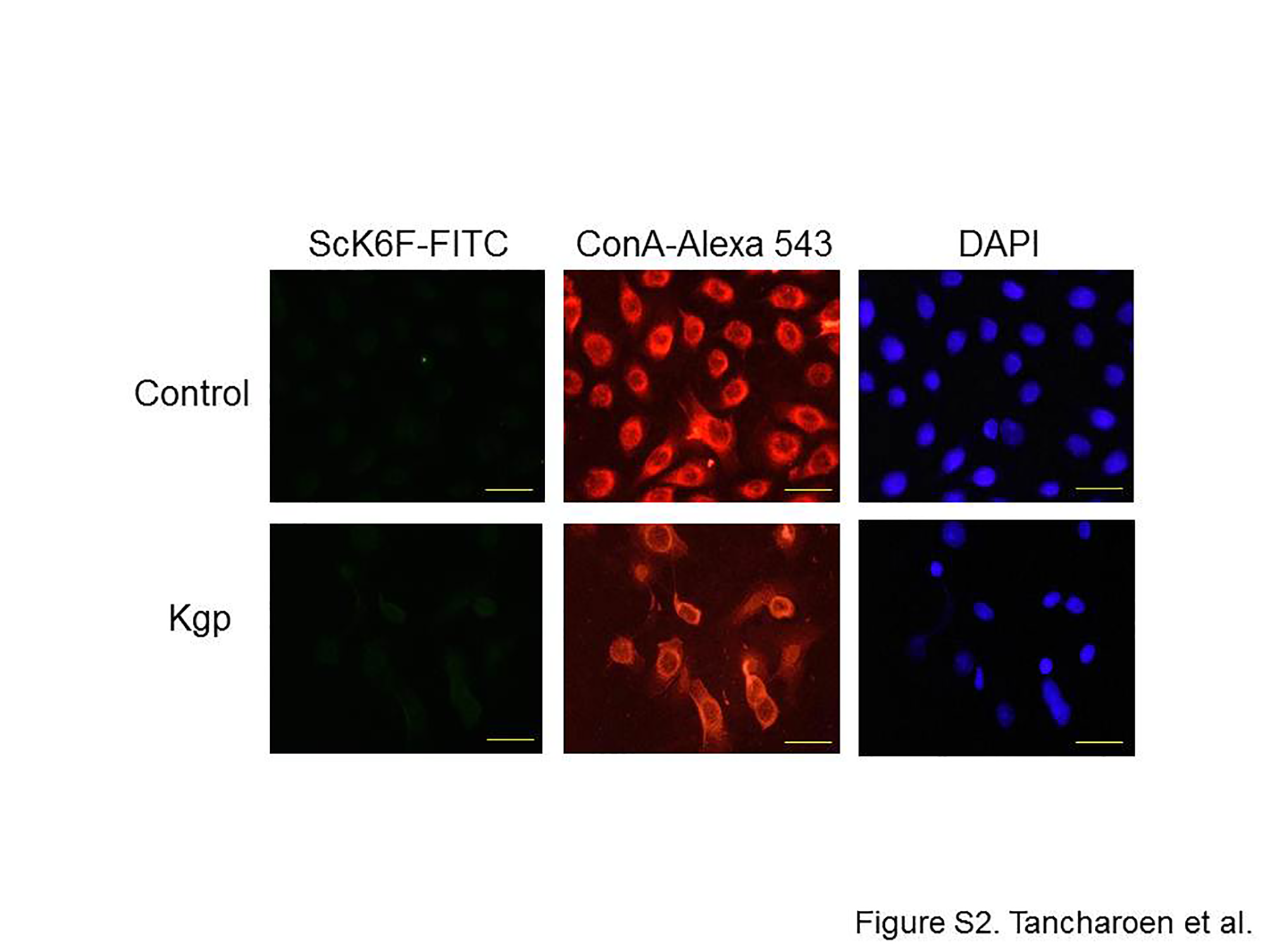

Supplement: S2 Fig — Rat GECs were incubated in the presence or absence of 50 nM Kgp for 6 h, double-stained using anti-ScK6F antibody (FITC, green) and ConA (Alexa543, red), and counter-stained for DNA with DAPI (blue). All images were obtained with a fluorescence microscope at ×400 magnification. Scale bar = 30 μm. (TIF) [file pone.0117775.s002.tif]

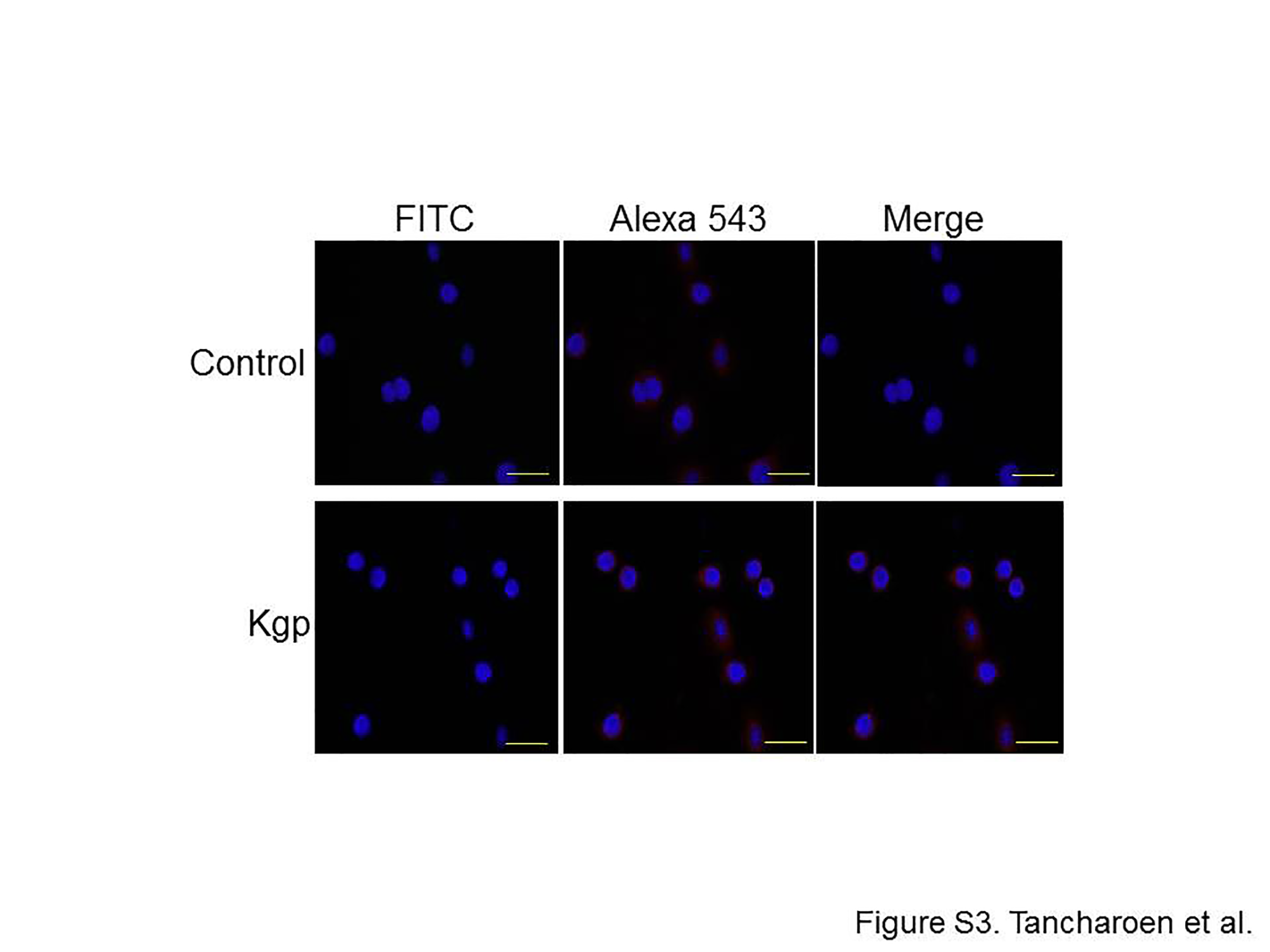

Supplement: S3 Fig — Rat GECs were incubated in the presence or absence of 50 nM Kgp for 6 h, double-stained using control rabbit IgG (FITC, green) and ConA (Alexa543, red), and counter-stained for DNA with DAPI (blue). All images were obtained with a fluorescence microscope at ×400 magnification. Scale bar = 30 μm. (TIF) [file pone.0117775.s003.tif]
